# Crystal structures of monohydrate and methanol solvate compounds of {1-[(3,5-bis{[(4,6-dimethylpyridin-2-yl)amino]methyl}-2,4,6-triethylbenzyl)amino]cyclopentyl}methanol

**DOI:** 10.1107/S2056989020012554

**Published:** 2020-09-25

**Authors:** Manuel Stapf, Wilhelm Seichter, Monika Mazik

**Affiliations:** a Technische Universität Bergakademie Freiberg, Leipziger Str. 29, D-09596 Freiberg/Sachsen, Germany

**Keywords:** crystal structures, tripodal mol­ecule, hydrogen bonding, C—H⋯π and π–π inter­actions

## Abstract

In the title monohydrate compound, **1a**, and the methanol solvate compound, **1b**, the tri­ethyl­benzene derivative, C_35_H_51_N_5_O, has three functionalized side arms and three ethyl groups, the former being located on one side of the central benzene ring, while the latter are directed to the opposite side. Both the crystals are constructed of structurally similar dimers of 1:1 host–guest complexes held together by N—H⋯O and O—H⋯N hydrogen bonds, and in **1a** additionally by O—H⋯O hydrogen bonds. The structure of **1b** contains additional highly disordered solvent mol­ecules. Thus, the SQUEEZE routine [Spek (2015 ▸). *Acta Cryst.* C**71**, 9–18] in *PLATON* was used to generate a modified data set, in which the contribution of the disordered mol­ecules to the structure amplitudes is eliminated. These solvent mol­ecules are not considered in the reported chemical formula.

## Abstract 2

In the crystal structures of the monohydrate and methanol solvate title compounds, the host mol­ecule adopts similar geometries with an alternating orientation of the substituents above and below the plane of the central arene ring.

## Chemical context   

Representatives of the class of 1,3,5-tris­ubstituted 2,4,6-tri­alkyl­benzenes have been shown to have the ability to act as artificial carbohydrate receptors. Depending on the nature of their building blocks, these compounds display different, inter­esting binding efficiencies and selectivities towards carbohydrates (Mazik, 2009 ▸, 2012 ▸; Stapf *et al.*, 2020 ▸). Our systematic studies have shown the enormous potential of this acyclic receptor architecture for versatile structural modifications, which enable the identification of inter­esting structure–activity relationships. For example, we have observed that the combination of two 2-amino­pyridine units with another recognition group provides receptors having a binding preference for *β*-glucoside *vs β*-galactoside (Mazik & Kuschel, 2008 ▸; Mazik & Geffert, 2011 ▸; Stapf *et al.*, 2020 ▸).
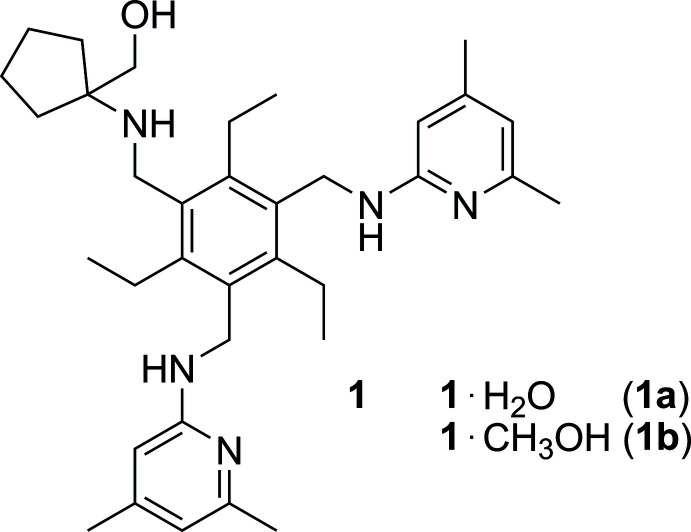


{1-[(3,5-Bis{[(4,6-dimethylpyridin-2-yl)amino]methyl}-2,4,6-triethylbenzyl)amino]cyclopentyl}methanol, **1**, represents a tri­ethyl­benzene derivative bearing, in addition to the above-mentioned pyridinyl units, a 1-hy­droxy­methyl-cyclo­pentyl­amino group. The crystal structures of the monohydrate, **1a**, and the methanol solvate, **1b**, are described here.

## Structural commentary   

**Figure 1:**
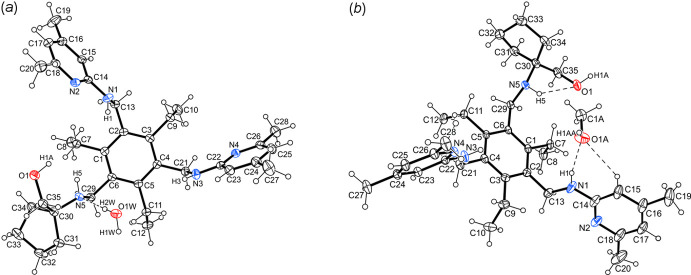
Perspective views of the structures of the 1:1 host–guest complexes, **1a** (*a*) and **1b** (*b*), including atom labelling. Anisotropic displacement ellipsoids are drawn at the 50% probability level.

Compounds **1a** and **1b** crystallize in the space groups *P*2_1_/*c* and *P* 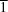, respectively. The mol­ecular structures depicted in Figs. 1 ▸ and 2 ▸ reveal similar host geometries with a fully alternating arrangement of the substituents above and below the plane of the central arene ring [*ab’ab’ab’* pattern, *a* = above, *b* = below (*a′*/*b′* = Et above/below); for a discussion on the conformations of tri­ethyl­benzene-based compounds, see: Koch *et al.*, 2017 ▸; Schulze *et al.*, 2017 ▸]. In other words, the three functionalized side arms point to one face of the central benzene ring and participate in the formation of hydrogen bonds with the guest solvent mol­ecule, while the ethyl groups are directed to the opposite side. The heterocyclic units are inclined by 62.4 (1) and 73.0 (1)° for **1a** [78.9 (1) and 85.1 (1)° for **1b**] with respect to the benzene ring. The cyclo­pentane rings adopt a slightly distorted envelope conformation with C33 (**1a**) and C31 (**1b**) as the flap.

## Supra­molecular features   

**Figure 3:**
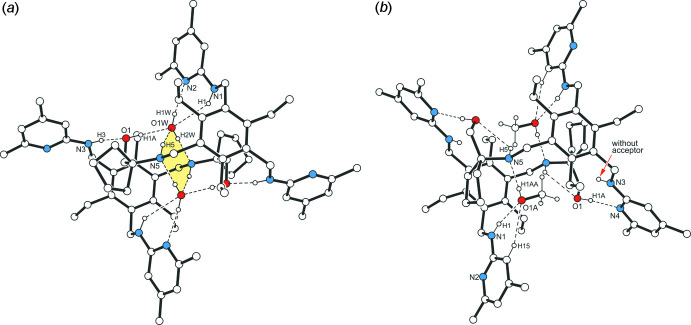
Supra­molecular motifs in the crystal structures of **1a** (*a*) and **1b** (*b*). For the sake of clarity, H atoms of the host mol­ecules not involved in hydrogen bonding are omitted.

**Figure 4:**
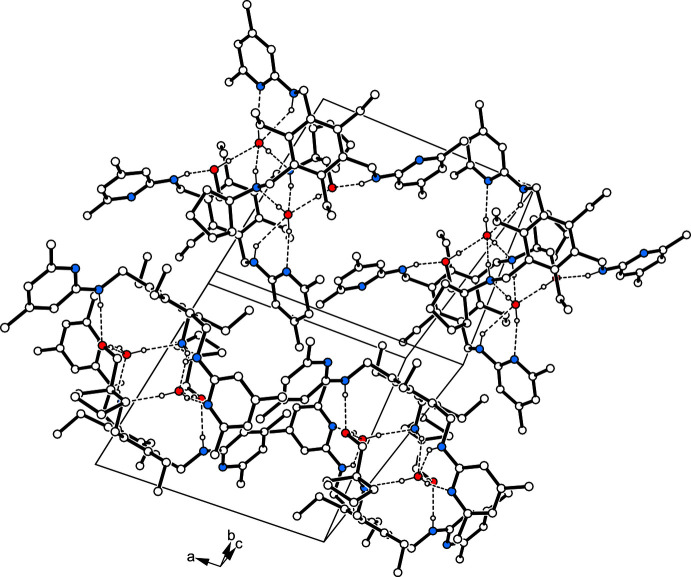
Packing diagram of **1a**. Dashed lines represent hydrogen bonds. H atoms not involved in the hydrogen bonds are omitted.

**Figure 5:**
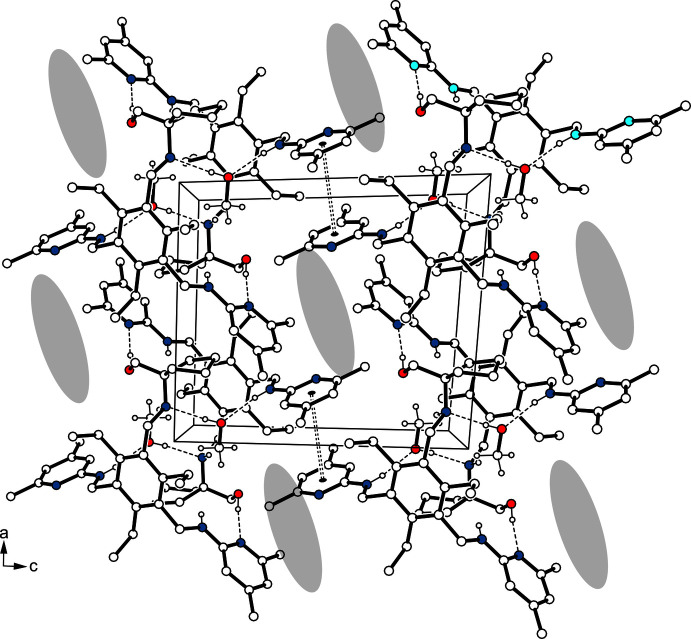
Packing diagram of **1b** viewed down the *b* axis. Dashed lines represent hydrogen bonds, dashed double lines π–π inter­actions. Areas occupied by disordered solvent mol­ecules are highlighted as grey ellipsoids. H atoms of the host mol­ecules not involved in the hydrogen bonds are omitted.

The crystal structures of **1a** and **1b** are composed of inversion-symmetric dimers of 1:1 host–guest complexes (Fig. 3 ▸). The donor/acceptor properties of the solvent species have, however, a marked influence on the patterns of hydrogen-bonding inter­actions. In the crystal of **1a**, the dimeric structural unit is held together by classical hydrogen bonds (N5—H5⋯O1*W* ^i^ and O1*W*—H2*W*⋯N5; symmetry code as given in Table 1 ▸) that contribute to the formation of a cyclic supra­molecular synthon with a graph-set motif 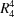
(8). Within this dimeric unit, the oxygen atom of the water mol­ecules acts as a trifurcated acceptor, as it is involved in the formation of an O—H⋯O bond [*d*(H⋯O) = 1.83 (1) Å] and two N_amine_—H⋯O inter­actions [*d*(H⋯O) = 2.50 (1) and 2.52 (1) Å]. The H atoms of the water mol­ecule participate in an asymmetric fashion in O—H⋯N bonding [*d*(H⋯N) = 2.03 (1) and 1.93 (1) Å] with pyridine atom N2 and amine atom N5, respectively, of different host mol­ecules. The inter­actions between the host mol­ecules are confined to only one N—H⋯O hydrogen bond [*d*(H⋯O) = 2.05 (1) Å] per mol­ecule. In the crystal of **1a**, the complexes are connected *via* C—H⋯π inter­actions [*d*(H⋯*Cg*) = 2.69 and 2.84 Å], forming a three-dimensional network. An portion of the crystal structure is displayed in Fig. 4 ▸. The presence of the alcohol solvent in **1b** reduces the number of hydrogen bonds within the dimeric structural unit (Table 2 ▸). In this case, the complex components create a continuous pattern of hydrogen bonds in the structure: N—H⋯O_solv_—H⋯N—H⋯O_host_—H⋯N_pyr_ [*d*(H⋯O) = 2.01 (3) and 2.36 (3) Å; *d*(H⋯N) = 1.90 and 1.97 Å]. While one of the amine hydrogen atoms is excluded from hydrogen bonding, a second one contributes by the formation of an intra­molecular N—H⋯O bond. Cross-linking of the complexes *via* C—H⋯π and π–π inter­actions [*Cg*2⋯*Cg*2^iv^ = 4.076 (2) Å; *Cg*2 is the centroid of the C14–C18/N2 ring; symmetry code: (iv) −*x* + 2, −*y* + 1, −*z* + 1] results in a three-dimensional supra­molecular architecture. A view of the crystal structure along the *b* axis reveals channel-like lattice voids in which the disordered solvent mol­ecules are accommodated (Fig. 5 ▸).

## Database survey   

A search in the Cambridge Structural Database (CSD, Version 5.40, update of February 2019; Groom *et al.*, 2016 ▸) for 1,3,5-tris­ubstituted 2,4,6-tri­alkyl­benzene derivatives containing the 4,6-di­methyl­pyridin-2-yl-amino­methyl subunit resulted in five hits. They include 1,3,5-tris­[(4,6-di­methyl­pyridin-2-yl)amino­meth­yl]-2,4,6-tri­methyl­benzene (refcode QAPVAF; Mazik *et al.*, 2005 ▸), which has proven to be an effective receptor for complex formation with methyl *β*-d-gluco­pyran­oside in the solid state, as well as the ethanol solvates of 1,3,5-tris­[(4,6-di­methyl­pyridin-2-yl)amino­meth­yl]-2,4,6-tri­methyl­benzene (RAJYUX; Mazik *et al.*, 2004 ▸) and of 1,3,5-tris­[(4,6-di­methyl­pyridin-2-yl)amino­meth­yl]-2,4,6-tri­ethyl­benzene (RAJZAE; Mazik *et al.*, 2004 ▸). In the crystals of the ethanol solvates (Mazik *et al.*, 2004 ▸), the functionalized side arms of the corresponding host are arranged in an *aab* fashion with respect to the benzene plane. In addition to the solvates of the symmetrical tris­ubstituted trimethyl- and tri­ethyl­benzene derivatives, the crystal structures of the solvates of two tri­ethyl­benzene-based compounds containing one or two phenanthrolinyl groups (ROKJEH, ROKJEH01; Mazik & Hartmann, 2008 ▸; Mazik *et al.*, 2009 ▸) in addition to the 2-amino­pyridine unit(s) have also been reported. In the case of 1-[*N*-(1,10-phenanthrolin-2-yl-carbon­yl)amino­meth­yl]-3,5-bis­[(4,6-di­methyl­pyridin-2-yl)amino­meth­yl]-2,4,6-tri­ethyl­ben­z­ene, three water mol­ecules are located in the binding pocket created by the heterocyclic units of the host (Mazik & Hartmann, 2008 ▸), whereas the binding pocket of 1,3-bis­[*N*-(1,10-phenanthrolin-2-yl-carbon­yl)amino­meth­yl]-5-[(4,6-di­methyl­pyridin-2-yl)amino­meth­yl]-2,4,6-tri­ethyl­benzene is filled with one ethanol and two water mol­ecules (Mazik *et al.*, 2009 ▸). The above-mentioned aggregates are stabilized by eight and ten hydrogen bonds, respectively.

## Synthesis and crystallization   

To a solution of 1-amino-1-cyclo­pentyl­methanol (1.48 mmol) in aceto­nitrile (20 ml) was added 1-bromo­methyl-3,5-bis­[(4,6-di­methyl­pyridin-2-yl)amino­methyl-2,4,6-tri­ethyl­benzene (0.71 mmol) dissolved in tetra­hydro­furan/aceto­nitrile (20 ml, 1:1 *v*/*v*). The reaction mixture was stirred at room temperature and under exclusion of light. The completion of the reaction was monitored by TLC. After evaporation of the solvents *in vacuo* and purification of the yellowish crude product *via* column chromatography (SiO_2_; chloro­form/methanol 7:1 *v*/*v*), compound **1** was obtained as a white solid. Yield: 33%; *R*_f_ = 0.26 (chloro­form/methanol 7:1 *v*/*v*); m.p. 375 K. Crystals of **1a** and **1b** suitable for single crystal X-ray diffraction were grown by slow evaporation of the respective solvent (aceto­nitrile in case of **1a**) at ambient temperature. ^1^H NMR (500 MHz, CDCl_3_, ppm) *δ* = 1.23 (*t+t*, 9H, *J* = 7.3 Hz), 1.55–1.65 (*m*, 4H), 1.68–1.77 (*m*, 4H), 2.23 (*s*, 6H), 2.36 (*s*, 6H), 2.70 (*q*, 2H, *J* = 7.3 Hz), 2.82 (*br*, 4H), 3.46 (*s*, 2H), 3.74 (*br*, 2H), 4.36 (*br*, 4H), 6.13 (*s*, 2H), 6.33 (*s*, 2H). ^13^C NMR (125 MHz, CDCl_3_, ppm) *δ* = 16.7, 16.9, 21.1, 22.9, 23.4, 23.9, 24.2, 33.5, 40.5 (2C), 64.7, 67.7, 103.8, 113.6, 132.8 (2C), 143.8 (2C), 148.8, 156.2, 158.2.

## Refinement   

Crystal data, data collection and structure refinement details are summarized in Table 3 ▸. N- and O-bound H atoms in **1a** were located in a difference-Fourier map and refined freely with distance restraints of N—H = 0.89 (1) Å and O—H = 0.85 (1) Å. For **1b**, N-bound H atoms were refined freely, while O-bound H atoms were treated as riding with O—H = 0.84 Å. All other H atoms were positioned geometrically and refined as riding, with C—H = 0.93–0.99 Å, and with *U*_iso_(H) = 1.5*U*_eq_(C) for methyl groups and *U*_iso_(H) = 1.2*U*_eq_(C) for other H atoms. The crystal structure **1b** contains highly disordered solvent mol­ecules that could not be refined to an acceptable level. Thus, the SQUEEZE routine (Spek, 2015 ▸) in the *PLATON* (Spek, 2020 ▸) program was used to generate a modified data set in which the contribution of the disordered mol­ecules to the structure amplitudes is eliminated. These solvent mol­ecules are not considered in the given chemical formula. The void volume of 267.9 Å^3^ occupied by the disordered solvent represents 14.3% of the cell volume, and the calculated electron count was 65 per void.

## Supplementary Material

Crystal structure: contains datablock(s) 1a, 1b, global. DOI: 10.1107/S2056989020012554/is5557sup1.cif

Structure factors: contains datablock(s) 1a. DOI: 10.1107/S2056989020012554/is55571asup2.hkl

Structure factors: contains datablock(s) 1b. DOI: 10.1107/S2056989020012554/is55571bsup3.hkl

Supporting information file. DOI: 10.1107/S2056989020012554/is55571asup4.cml

Supporting information file. DOI: 10.1107/S2056989020012554/is55571bsup5.cml

CCDC references: 2032104, 2032103

Additional supporting information:  crystallographic information; 3D view; checkCIF report

## Figures and Tables

**Figure 2:**
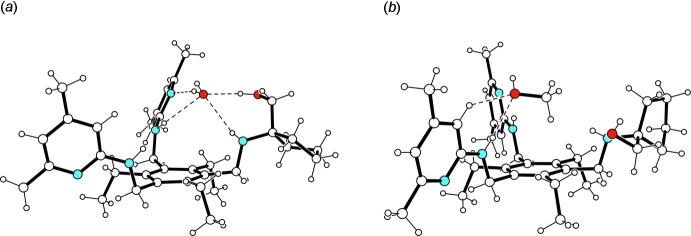
Ball-and-stick representations (side views) of the 1:1 host–guest complexes, **1a** (*a*) and **1b** (*b*).

**Table 1:** Hydrogen-bond geometry (Å, °) for **1a** [Chem scheme1] *Cg*1 and *Cg*2 are the centroids of the C1–C6 and C14–C18/N2 rings, respectively.

*D*—H⋯*A*	*D*—H	H⋯*A*	*D*⋯*A*	*D*—H⋯*A*
O1*W*—H2*W*⋯N5	0.87 (1)	1.93 (1)	2.8018 (14)	175 (2)
O1*W*—H1*W*⋯N2^i^	0.86 (1)	2.03 (1)	2.8780 (15)	166 (2)
O1—H1*A*⋯O1*W* ^i^	0.87 (1)	1.83 (1)	2.6993 (14)	176 (2)
N5—H5⋯O1*W* ^i^	0.90 (1)	2.52 (1)	3.3302 (14)	151 (1)
N3—H3⋯O1^i^	0.89 (1)	2.05 (1)	2.9115 (15)	162 (2)
N1—H1⋯O1*W* ^i^	0.89 (1)	2.50 (1)	3.2618 (16)	145 (1)
C32—H32*B*⋯*Cg*1^ii^	0.99	2.69	3.666 (2)	169
C25—H25⋯*Cg*2^iii^	0.95	2.84	3.728 (2)	156

Symmetry codes: (i) 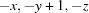; (ii) 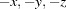; (iii) 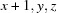.

**Table 2:** Hydrogen-bond geometry (Å, °) for **1b** [Chem scheme1] *Cg*1 and *Cg*3 are the centroids of the C1–C6 and C22–C26/N4 rings, respectively.

*D*—H⋯*A*	*D*—H	H⋯*A*	*D*⋯*A*	*D*—H⋯*A*
N1—H1⋯O1*A*	0.94 (3)	2.01 (3)	2.930 (3)	162 (2)
C15—H15⋯O1*A*	0.95	2.56	3.318 (3)	137
N5—H5⋯O1	0.90 (3)	2.36 (3)	2.823 (3)	112 (2)
O1—H1*A*⋯N4^i^	0.84	1.90	2.741 (3)	174
O1*A*—H1*AA*⋯N5^i^	0.84	1.97	2.798 (3)	170
C27—H27*A*⋯*Cg*1^ii^	0.98	2.67	3.541 (3)	148
C32—H32*B*⋯*Cg*3^iii^	0.98	2.69	3.614 (3)	156

Symmetry codes: (i) 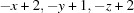; (ii) 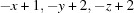; (iii) 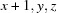.

**Table 3:** Experimental details

	**1b**	**1b**
Crystal data		
Chemical formula	C_35_H_51_N_5_O·H_2_O	C_35_H_51_N_5_O·CH_4_O
*M*_r_	575.82	589.85
Crystal system, space group	Monoclinic, *P*2_1_/*c*	Triclinic, *P* 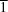
Temperature (K)	100	100
*a*, *b*, *c* (Å)	13.2239 (7), 15.3576 (8), 17.1061 (8)	12.1169 (4), 13.2380 (5), 13.6258 (5)
α, β, γ (°)	90, 107.0289 (17), 90	68.373 (2), 79.379 (2), 67.392 (2)
*V* (Å^3^)	3321.7 (3)	1873.32 (12)
*Z*	4	2
Radiation type	Mo *K*α	Mo *K*α
μ (mm^−1^)	0.07	0.07
Crystal size (mm)	0.45 × 0.32 × 0.10	0.56 × 0.34 × 0.32

Data collection		
Diffractometer	Bruker X8 APEXII CCD	Bruker X8 APEXII CCD
No. of measured, independent and observed [*I* > 2σ(*I*)] reflections	28794, 7467, 5791	32191, 7654, 5160
*R*_int_	0.034	0.022
(sin θ/λ)_max_ (Å^−1^)	0.647	0.626

Refinement		
*R*[*F* ^2^ > 2σ(*F* ^2^)], *wR*(*F* ^2^), *S*	0.043, 0.114, 1.03	0.065, 0.184, 1.21
No. of reflections	7467	7654
No. of parameters	410	410
No. of restraints	6	0
H-atom treatment	H atoms treated by a mixture of independent and constrained refinement	H atoms treated by a mixture of independent and constrained refinement
Δρ_max_, Δρ_min_ (e Å^−3^)	0.27, −0.20	0.64, −0.39

Computer programs: *APEX2* and *SAINT* (Bruker, 2014 ▸), *SHELXS97* and *SHELXTL* (Sheldrick, 2008 ▸), *SHELXL2014* (Sheldrick, 2015 ▸) and *ORTEP-3 for Windows* (Farrugia, 2012 ▸).

## supplementary crystallographic information

### {1-[(3,5-Bis{[(4,6-dimethylpyridin-2-yl)amino]methyl}-2,4,6-triethylbenzyl)amino]cyclopentyl}methanol monohydrate (1a) Crystal data

**Table:**

C_35_H_51_N_5_O·H_2_O	*F*(000) = 1256
*M**_r_* = 575.82	*D*_x_ = 1.151 Mg m^−^^3^
Monoclinic, *P*2_1_/*c*	Mo *K*α radiation, λ = 0.71073 Å
*a* = 13.2239 (7) Å	Cell parameters from 9674 reflections
*b* = 15.3576 (8) Å	θ = 2.2–28.2°
*c* = 17.1061 (8) Å	µ = 0.07 mm^−^^1^
β = 107.0289 (17)°	*T* = 100 K
*V* = 3321.7 (3) Å^3^	Irregular, colourless
*Z* = 4	0.45 × 0.32 × 0.10 mm

### {1-[(3,5-Bis{[(4,6-dimethylpyridin-2-yl)amino]methyl}-2,4,6-triethylbenzyl)amino]cyclopentyl}methanol monohydrate (1a) Data collection

**Table:**

Bruker X8 APEXII CCD diffractometer	*R*_int_ = 0.034
phi and ω scans	θ_max_ = 27.4°, θ_min_ = 2.1°
28794 measured reflections	*h* = −17→17
7467 independent reflections	*k* = −19→17
5791 reflections with *I* > 2σ(*I*)	*l* = −22→21

### {1-[(3,5-Bis{[(4,6-dimethylpyridin-2-yl)amino]methyl}-2,4,6-triethylbenzyl)amino]cyclopentyl}methanol monohydrate (1a) Refinement

**Table:**

Refinement on *F*^2^	6 restraints
Least-squares matrix: full	Hydrogen site location: mixed
*R*[*F*^2^ > 2σ(*F*^2^)] = 0.043	H atoms treated by a mixture of independent and constrained refinement
*wR*(*F*^2^) = 0.114	*w* = 1/[σ^2^(*F*_o_^2^) + (0.0551*P*)^2^ + 0.8757*P*] where *P* = (*F*_o_^2^ + 2*F*_c_^2^)/3
*S* = 1.02	(Δ/σ)_max_ < 0.001
7467 reflections	Δρ_max_ = 0.27 e Å^−^^3^
410 parameters	Δρ_min_ = −0.20 e Å^−^^3^

### {1-[(3,5-Bis{[(4,6-dimethylpyridin-2-yl)amino]methyl}-2,4,6-triethylbenzyl)amino]cyclopentyl}methanol monohydrate (1a) Special details

**Table:**

Geometry. All esds (except the esd in the dihedral angle between two l.s. planes) are estimated using the full covariance matrix. The cell esds are taken into account individually in the estimation of esds in distances, angles and torsion angles; correlations between esds in cell parameters are only used when they are defined by crystal symmetry. An approximate (isotropic) treatment of cell esds is used for estimating esds involving l.s. planes.

### {1-[(3,5-Bis{[(4,6-dimethylpyridin-2-yl)amino]methyl}-2,4,6-triethylbenzyl)amino]cyclopentyl}methanol monohydrate (1a) Fractional atomic coordinates and isotropic or equivalent isotropic displacement parameters (Å^2^)

**Table:**

	*x*	*y*	*z*	*U*_iso_*/*U*_eq_	
O1	−0.21807 (7)	0.54810 (6)	0.00959 (6)	0.0238 (2)	
N1	−0.11044 (9)	0.24557 (8)	−0.12255 (7)	0.0218 (2)	
H1	−0.0834 (13)	0.2969 (7)	−0.1039 (10)	0.036 (5)*	
N2	−0.23912 (9)	0.33295 (7)	−0.20586 (6)	0.0198 (2)	
N3	0.33881 (9)	0.29584 (8)	0.05481 (7)	0.0223 (3)	
H3	0.3046 (12)	0.3464 (7)	0.0466 (10)	0.036 (5)*	
N4	0.44662 (8)	0.20100 (7)	0.00817 (7)	0.0201 (2)	
N5	−0.03784 (8)	0.45806 (7)	0.12196 (6)	0.0176 (2)	
H5	−0.0752 (11)	0.4402 (10)	0.0720 (6)	0.024 (4)*	
C1	−0.03521 (10)	0.26699 (8)	0.06516 (7)	0.0163 (3)	
C2	−0.00019 (10)	0.21235 (8)	0.01228 (7)	0.0161 (3)	
C3	0.10826 (10)	0.20103 (8)	0.02240 (7)	0.0167 (3)	
C4	0.18206 (10)	0.24315 (8)	0.08766 (7)	0.0169 (3)	
C5	0.14833 (10)	0.30214 (8)	0.13754 (7)	0.0172 (3)	
C6	0.03906 (10)	0.31377 (8)	0.12622 (7)	0.0166 (3)	
C7	−0.15304 (10)	0.27362 (9)	0.05636 (8)	0.0197 (3)	
H7A	−0.1936	0.2693	−0.0022	0.024*	
H7B	−0.1682	0.3311	0.0766	0.024*	
C8	−0.18900 (11)	0.20182 (10)	0.10409 (9)	0.0264 (3)	
H8A	−0.1753	0.1448	0.0835	0.040*	
H8B	−0.2649	0.2080	0.0971	0.040*	
H8C	−0.1499	0.2066	0.1622	0.040*	
C9	0.14599 (11)	0.14569 (9)	−0.03707 (8)	0.0223 (3)	
H9A	0.2149	0.1684	−0.0399	0.027*	
H9B	0.0949	0.1512	−0.0922	0.027*	
C10	0.15839 (13)	0.04917 (10)	−0.01346 (10)	0.0335 (4)	
H10A	0.2087	0.0431	0.0412	0.050*	
H10B	0.1847	0.0173	−0.0532	0.050*	
H10C	0.0897	0.0254	−0.0133	0.050*	
C11	0.22863 (10)	0.35482 (9)	0.20189 (8)	0.0215 (3)	
H11A	0.1992	0.4135	0.2052	0.026*	
H11B	0.2932	0.3620	0.1845	0.026*	
C12	0.25915 (12)	0.31327 (10)	0.28712 (8)	0.0288 (3)	
H12A	0.1950	0.3008	0.3028	0.043*	
H12B	0.3042	0.3535	0.3268	0.043*	
H12C	0.2977	0.2590	0.2863	0.043*	
C13	−0.08201 (10)	0.17532 (9)	−0.06179 (8)	0.0190 (3)	
H13A	−0.0524	0.1251	−0.0840	0.023*	
H13B	−0.1451	0.1556	−0.0468	0.023*	
C14	−0.21009 (10)	0.25244 (9)	−0.17714 (7)	0.0181 (3)	
C15	−0.27565 (10)	0.17971 (9)	−0.20134 (8)	0.0206 (3)	
H15	−0.2528	0.1238	−0.1796	0.025*	
C16	−0.37413 (11)	0.19050 (9)	−0.25737 (9)	0.0246 (3)	
C17	−0.40303 (11)	0.27384 (9)	−0.28877 (8)	0.0237 (3)	
H17	−0.4696	0.2832	−0.3282	0.028*	
C18	−0.33386 (10)	0.34256 (9)	−0.26202 (8)	0.0205 (3)	
C19	−0.44869 (13)	0.11508 (11)	−0.28255 (12)	0.0432 (4)	
H19A	−0.4083	0.0607	−0.2750	0.065*	
H19B	−0.4893	0.1213	−0.3402	0.065*	
H19C	−0.4972	0.1140	−0.2489	0.065*	
C20	−0.36075 (12)	0.43342 (10)	−0.29491 (9)	0.0282 (3)	
H20A	−0.3585	0.4732	−0.2497	0.042*	
H20B	−0.4319	0.4338	−0.3338	0.042*	
H20C	−0.3094	0.4523	−0.3227	0.042*	
C21	0.29964 (10)	0.22967 (9)	0.09957 (8)	0.0201 (3)	
H21A	0.3387	0.2332	0.1585	0.024*	
H21B	0.3115	0.1711	0.0797	0.024*	
C22	0.40895 (10)	0.28164 (9)	0.01095 (8)	0.0191 (3)	
C23	0.44048 (11)	0.35362 (9)	−0.02740 (8)	0.0227 (3)	
H23	0.4112	0.4096	−0.0245	0.027*	
C24	0.51400 (11)	0.34216 (9)	−0.06903 (9)	0.0249 (3)	
C25	0.55286 (11)	0.25789 (9)	−0.07272 (9)	0.0241 (3)	
H25	0.6034	0.2474	−0.1013	0.029*	
C26	0.51739 (10)	0.19036 (9)	−0.03474 (8)	0.0217 (3)	
C27	0.55221 (14)	0.41751 (11)	−0.10917 (11)	0.0386 (4)	
H27A	0.5240	0.4122	−0.1687	0.058*	
H27B	0.6297	0.4171	−0.0935	0.058*	
H27C	0.5278	0.4723	−0.0914	0.058*	
C28	0.55646 (13)	0.09887 (10)	−0.03838 (10)	0.0306 (3)	
H28A	0.5931	0.0787	0.0170	0.046*	
H28B	0.6054	0.0978	−0.0717	0.046*	
H28C	0.4963	0.0605	−0.0629	0.046*	
C29	−0.00203 (10)	0.38089 (8)	0.17448 (8)	0.0190 (3)	
H29A	−0.0616	0.3563	0.1914	0.023*	
H29B	0.0547	0.3976	0.2243	0.023*	
C30	−0.10008 (10)	0.52576 (8)	0.14971 (8)	0.0192 (3)	
C31	−0.02821 (11)	0.57698 (9)	0.22365 (8)	0.0239 (3)	
H31A	0.0443	0.5526	0.2400	0.029*	
H31B	−0.0245	0.6391	0.2094	0.029*	
C32	−0.07871 (14)	0.56730 (11)	0.29311 (9)	0.0360 (4)	
H32A	−0.0488	0.5167	0.3281	0.043*	
H32B	−0.0677	0.6204	0.3274	0.043*	
C33	−0.19561 (13)	0.55366 (10)	0.24928 (10)	0.0341 (4)	
H33A	−0.2308	0.6094	0.2284	0.041*	
H33B	−0.2323	0.5265	0.2859	0.041*	
C34	−0.19394 (11)	0.49275 (9)	0.17924 (8)	0.0230 (3)	
H34A	−0.2613	0.4962	0.1346	0.028*	
H34B	−0.1823	0.4317	0.1985	0.028*	
C35	−0.14183 (11)	0.58756 (9)	0.07718 (8)	0.0218 (3)	
H35A	−0.1743	0.6388	0.0954	0.026*	
H35B	−0.0816	0.6084	0.0589	0.026*	
O1W	0.12410 (8)	0.54814 (6)	0.08143 (6)	0.0228 (2)	
H1A	−0.1888 (14)	0.5189 (11)	−0.0219 (10)	0.053 (6)*	
H1W	0.1623 (13)	0.5760 (11)	0.1234 (8)	0.049 (6)*	
H2W	0.0765 (13)	0.5196 (12)	0.0970 (12)	0.056 (6)*	

### {1-[(3,5-Bis{[(4,6-dimethylpyridin-2-yl)amino]methyl}-2,4,6-triethylbenzyl)amino]cyclopentyl}methanol monohydrate (1a) Atomic displacement parameters (Å^2^)

**Table:**

	*U*^11^	*U*^22^	*U*^33^	*U*^12^	*U*^13^	*U*^23^
O1	0.0248 (5)	0.0247 (5)	0.0211 (5)	0.0028 (4)	0.0057 (4)	−0.0022 (4)
N1	0.0216 (6)	0.0227 (6)	0.0174 (5)	−0.0049 (5)	0.0001 (5)	0.0022 (5)
N2	0.0228 (6)	0.0218 (6)	0.0145 (5)	−0.0014 (4)	0.0050 (4)	−0.0001 (5)
N3	0.0219 (6)	0.0215 (6)	0.0262 (6)	0.0011 (5)	0.0114 (5)	0.0022 (5)
N4	0.0202 (5)	0.0211 (6)	0.0181 (5)	−0.0012 (4)	0.0044 (4)	0.0001 (5)
N5	0.0225 (5)	0.0153 (5)	0.0154 (5)	0.0005 (4)	0.0063 (4)	−0.0017 (4)
C1	0.0194 (6)	0.0137 (6)	0.0158 (6)	0.0003 (5)	0.0052 (5)	0.0049 (5)
C2	0.0203 (6)	0.0123 (6)	0.0143 (6)	−0.0016 (5)	0.0031 (5)	0.0026 (5)
C3	0.0225 (6)	0.0141 (6)	0.0145 (6)	0.0010 (5)	0.0069 (5)	0.0029 (5)
C4	0.0181 (6)	0.0172 (6)	0.0150 (6)	0.0002 (5)	0.0045 (5)	0.0045 (5)
C5	0.0207 (6)	0.0165 (6)	0.0135 (6)	−0.0013 (5)	0.0036 (5)	0.0025 (5)
C6	0.0213 (6)	0.0145 (6)	0.0145 (6)	0.0000 (5)	0.0060 (5)	0.0016 (5)
C7	0.0196 (6)	0.0179 (6)	0.0214 (7)	0.0000 (5)	0.0058 (5)	−0.0004 (5)
C8	0.0254 (7)	0.0261 (8)	0.0303 (8)	−0.0017 (6)	0.0121 (6)	0.0027 (6)
C9	0.0238 (7)	0.0241 (7)	0.0199 (7)	0.0007 (5)	0.0079 (5)	−0.0034 (6)
C10	0.0371 (8)	0.0227 (8)	0.0425 (9)	0.0041 (6)	0.0144 (7)	−0.0069 (7)
C11	0.0207 (6)	0.0236 (7)	0.0189 (6)	−0.0018 (5)	0.0037 (5)	−0.0033 (6)
C12	0.0301 (7)	0.0345 (8)	0.0177 (7)	−0.0014 (6)	0.0004 (6)	−0.0021 (6)
C13	0.0215 (6)	0.0177 (6)	0.0160 (6)	−0.0013 (5)	0.0029 (5)	−0.0004 (5)
C14	0.0196 (6)	0.0229 (7)	0.0126 (6)	0.0000 (5)	0.0059 (5)	−0.0015 (5)
C15	0.0232 (7)	0.0178 (7)	0.0198 (6)	0.0012 (5)	0.0045 (5)	−0.0040 (5)
C16	0.0238 (7)	0.0239 (7)	0.0235 (7)	−0.0014 (6)	0.0028 (6)	−0.0058 (6)
C17	0.0199 (6)	0.0283 (8)	0.0197 (7)	0.0018 (5)	0.0009 (5)	−0.0020 (6)
C18	0.0229 (6)	0.0239 (7)	0.0153 (6)	0.0018 (5)	0.0068 (5)	−0.0001 (5)
C19	0.0332 (8)	0.0283 (9)	0.0527 (10)	−0.0068 (7)	−0.0115 (8)	−0.0018 (8)
C20	0.0313 (8)	0.0271 (8)	0.0244 (7)	0.0014 (6)	0.0052 (6)	0.0053 (6)
C21	0.0192 (6)	0.0227 (7)	0.0180 (6)	0.0015 (5)	0.0048 (5)	0.0018 (5)
C22	0.0169 (6)	0.0230 (7)	0.0160 (6)	−0.0008 (5)	0.0024 (5)	−0.0014 (5)
C23	0.0255 (7)	0.0195 (7)	0.0238 (7)	0.0014 (5)	0.0084 (6)	0.0003 (6)
C24	0.0278 (7)	0.0245 (7)	0.0241 (7)	−0.0009 (6)	0.0101 (6)	0.0014 (6)
C25	0.0244 (7)	0.0266 (7)	0.0239 (7)	0.0020 (6)	0.0113 (6)	−0.0018 (6)
C26	0.0232 (7)	0.0229 (7)	0.0183 (6)	0.0009 (5)	0.0048 (5)	−0.0025 (6)
C27	0.0504 (10)	0.0276 (8)	0.0482 (10)	0.0014 (7)	0.0308 (9)	0.0057 (7)
C28	0.0370 (8)	0.0240 (8)	0.0341 (8)	0.0038 (6)	0.0158 (7)	0.0005 (6)
C29	0.0222 (6)	0.0186 (7)	0.0161 (6)	0.0013 (5)	0.0056 (5)	0.0007 (5)
C30	0.0229 (6)	0.0160 (6)	0.0200 (6)	0.0002 (5)	0.0081 (5)	−0.0019 (5)
C31	0.0301 (7)	0.0187 (7)	0.0226 (7)	−0.0014 (5)	0.0073 (6)	−0.0044 (6)
C32	0.0512 (10)	0.0353 (9)	0.0255 (8)	−0.0077 (8)	0.0172 (7)	−0.0105 (7)
C33	0.0475 (9)	0.0234 (8)	0.0418 (9)	−0.0019 (7)	0.0294 (8)	−0.0045 (7)
C34	0.0253 (7)	0.0215 (7)	0.0252 (7)	−0.0002 (5)	0.0119 (6)	−0.0005 (6)
C35	0.0258 (7)	0.0175 (7)	0.0227 (7)	0.0005 (5)	0.0080 (6)	−0.0011 (5)
O1W	0.0275 (5)	0.0216 (5)	0.0202 (5)	−0.0052 (4)	0.0084 (4)	−0.0029 (4)

### {1-[(3,5-Bis{[(4,6-dimethylpyridin-2-yl)amino]methyl}-2,4,6-triethylbenzyl)amino]cyclopentyl}methanol monohydrate (1a) Geometric parameters (Å, º)

**Table:**

O1—C35	1.4275 (16)	C15—H15	0.9500
O1—H1A	0.874 (9)	C16—C17	1.397 (2)
N1—C14	1.3779 (16)	C16—C19	1.500 (2)
N1—C13	1.4685 (17)	C17—C18	1.3837 (19)
N1—H1	0.885 (9)	C17—H17	0.9500
N2—C14	1.3443 (17)	C18—C20	1.5076 (19)
N2—C18	1.3451 (17)	C19—H19A	0.9800
N3—C22	1.3705 (17)	C19—H19B	0.9800
N3—C21	1.4558 (17)	C19—H19C	0.9800
N3—H3	0.889 (9)	C20—H20A	0.9800
N4—C22	1.3410 (17)	C20—H20B	0.9800
N4—C26	1.3578 (17)	C20—H20C	0.9800
N5—C29	1.4787 (16)	C21—H21A	0.9900
N5—C30	1.4876 (16)	C21—H21B	0.9900
N5—H5	0.896 (9)	C22—C23	1.4093 (19)
C1—C6	1.4039 (17)	C23—C24	1.3744 (19)
C1—C2	1.4080 (18)	C23—H23	0.9500
C1—C7	1.5243 (17)	C24—C25	1.401 (2)
C2—C3	1.4041 (17)	C24—C27	1.506 (2)
C2—C13	1.5155 (17)	C25—C26	1.377 (2)
C3—C4	1.4067 (18)	C25—H25	0.9500
C3—C9	1.5177 (18)	C26—C28	1.5046 (19)
C4—C5	1.4037 (18)	C27—H27A	0.9800
C4—C21	1.5219 (17)	C27—H27B	0.9800
C5—C6	1.4120 (17)	C27—H27C	0.9800
C5—C11	1.5197 (17)	C28—H28A	0.9800
C6—C29	1.5172 (18)	C28—H28B	0.9800
C7—C8	1.5284 (19)	C28—H28C	0.9800
C7—H7A	0.9900	C29—H29A	0.9900
C7—H7B	0.9900	C29—H29B	0.9900
C8—H8A	0.9800	C30—C35	1.5310 (18)
C8—H8B	0.9800	C30—C31	1.5548 (18)
C8—H8C	0.9800	C30—C34	1.5556 (18)
C9—C10	1.532 (2)	C31—C32	1.531 (2)
C9—H9A	0.9900	C31—H31A	0.9900
C9—H9B	0.9900	C31—H31B	0.9900
C10—H10A	0.9800	C32—C33	1.522 (2)
C10—H10B	0.9800	C32—H32A	0.9900
C10—H10C	0.9800	C32—H32B	0.9900
C11—C12	1.5333 (19)	C33—C34	1.525 (2)
C11—H11A	0.9900	C33—H33A	0.9900
C11—H11B	0.9900	C33—H33B	0.9900
C12—H12A	0.9800	C34—H34A	0.9900
C12—H12B	0.9800	C34—H34B	0.9900
C12—H12C	0.9800	C35—H35A	0.9900
C13—H13A	0.9900	C35—H35B	0.9900
C13—H13B	0.9900	O1W—H1W	0.861 (9)
C14—C15	1.4001 (18)	O1W—H2W	0.871 (9)
C15—C16	1.3824 (18)		
			
C35—O1—H1A	112.5 (13)	C16—C19—H19B	109.5
C14—N1—C13	122.12 (11)	H19A—C19—H19B	109.5
C14—N1—H1	112.4 (11)	C16—C19—H19C	109.5
C13—N1—H1	114.0 (11)	H19A—C19—H19C	109.5
C14—N2—C18	117.94 (11)	H19B—C19—H19C	109.5
C22—N3—C21	125.38 (12)	C18—C20—H20A	109.5
C22—N3—H3	116.6 (11)	C18—C20—H20B	109.5
C21—N3—H3	116.7 (11)	H20A—C20—H20B	109.5
C22—N4—C26	116.90 (11)	C18—C20—H20C	109.5
C29—N5—C30	118.35 (10)	H20A—C20—H20C	109.5
C29—N5—H5	108.8 (10)	H20B—C20—H20C	109.5
C30—N5—H5	108.4 (10)	N3—C21—C4	109.97 (10)
C6—C1—C2	119.53 (11)	N3—C21—H21A	109.7
C6—C1—C7	120.77 (11)	C4—C21—H21A	109.7
C2—C1—C7	119.70 (11)	N3—C21—H21B	109.7
C3—C2—C1	120.72 (11)	C4—C21—H21B	109.7
C3—C2—C13	120.66 (11)	H21A—C21—H21B	108.2
C1—C2—C13	118.17 (11)	N4—C22—N3	118.97 (12)
C2—C3—C4	119.15 (11)	N4—C22—C23	122.97 (12)
C2—C3—C9	120.74 (11)	N3—C22—C23	118.03 (12)
C4—C3—C9	120.10 (11)	C24—C23—C22	119.43 (13)
C5—C4—C3	120.60 (11)	C24—C23—H23	120.3
C5—C4—C21	120.03 (11)	C22—C23—H23	120.3
C3—C4—C21	119.19 (11)	C23—C24—C25	117.79 (13)
C4—C5—C6	119.53 (11)	C23—C24—C27	121.36 (13)
C4—C5—C11	120.33 (11)	C25—C24—C27	120.85 (13)
C6—C5—C11	120.12 (11)	C26—C25—C24	119.63 (12)
C1—C6—C5	120.14 (11)	C26—C25—H25	120.2
C1—C6—C29	117.93 (11)	C24—C25—H25	120.2
C5—C6—C29	121.78 (11)	N4—C26—C25	123.26 (12)
C1—C7—C8	111.59 (11)	N4—C26—C28	115.73 (12)
C1—C7—H7A	109.3	C25—C26—C28	121.01 (12)
C8—C7—H7A	109.3	C24—C27—H27A	109.5
C1—C7—H7B	109.3	C24—C27—H27B	109.5
C8—C7—H7B	109.3	H27A—C27—H27B	109.5
H7A—C7—H7B	108.0	C24—C27—H27C	109.5
C7—C8—H8A	109.5	H27A—C27—H27C	109.5
C7—C8—H8B	109.5	H27B—C27—H27C	109.5
H8A—C8—H8B	109.5	C26—C28—H28A	109.5
C7—C8—H8C	109.5	C26—C28—H28B	109.5
H8A—C8—H8C	109.5	H28A—C28—H28B	109.5
H8B—C8—H8C	109.5	C26—C28—H28C	109.5
C3—C9—C10	113.32 (11)	H28A—C28—H28C	109.5
C3—C9—H9A	108.9	H28B—C28—H28C	109.5
C10—C9—H9A	108.9	N5—C29—C6	108.41 (10)
C3—C9—H9B	108.9	N5—C29—H29A	110.0
C10—C9—H9B	108.9	C6—C29—H29A	110.0
H9A—C9—H9B	107.7	N5—C29—H29B	110.0
C9—C10—H10A	109.5	C6—C29—H29B	110.0
C9—C10—H10B	109.5	H29A—C29—H29B	108.4
H10A—C10—H10B	109.5	N5—C30—C35	106.05 (10)
C9—C10—H10C	109.5	N5—C30—C31	110.47 (11)
H10A—C10—H10C	109.5	C35—C30—C31	109.58 (11)
H10B—C10—H10C	109.5	N5—C30—C34	116.39 (11)
C5—C11—C12	113.62 (11)	C35—C30—C34	109.43 (11)
C5—C11—H11A	108.8	C31—C30—C34	104.86 (10)
C12—C11—H11A	108.8	C32—C31—C30	106.29 (11)
C5—C11—H11B	108.8	C32—C31—H31A	110.5
C12—C11—H11B	108.8	C30—C31—H31A	110.5
H11A—C11—H11B	107.7	C32—C31—H31B	110.5
C11—C12—H12A	109.5	C30—C31—H31B	110.5
C11—C12—H12B	109.5	H31A—C31—H31B	108.7
H12A—C12—H12B	109.5	C33—C32—C31	104.00 (12)
C11—C12—H12C	109.5	C33—C32—H32A	111.0
H12A—C12—H12C	109.5	C31—C32—H32A	111.0
H12B—C12—H12C	109.5	C33—C32—H32B	111.0
N1—C13—C2	106.75 (10)	C31—C32—H32B	111.0
N1—C13—H13A	110.4	H32A—C32—H32B	109.0
C2—C13—H13A	110.4	C32—C33—C34	103.01 (12)
N1—C13—H13B	110.4	C32—C33—H33A	111.2
C2—C13—H13B	110.4	C34—C33—H33A	111.2
H13A—C13—H13B	108.6	C32—C33—H33B	111.2
N2—C14—N1	115.60 (11)	C34—C33—H33B	111.2
N2—C14—C15	122.67 (12)	H33A—C33—H33B	109.1
N1—C14—C15	121.72 (12)	C33—C34—C30	104.58 (11)
C16—C15—C14	119.04 (13)	C33—C34—H34A	110.8
C16—C15—H15	120.5	C30—C34—H34A	110.8
C14—C15—H15	120.5	C33—C34—H34B	110.8
C15—C16—C17	118.19 (12)	C30—C34—H34B	110.8
C15—C16—C19	120.87 (13)	H34A—C34—H34B	108.9
C17—C16—C19	120.93 (13)	O1—C35—C30	113.25 (11)
C18—C17—C16	119.44 (12)	O1—C35—H35A	108.9
C18—C17—H17	120.3	C30—C35—H35A	108.9
C16—C17—H17	120.3	O1—C35—H35B	108.9
N2—C18—C17	122.67 (13)	C30—C35—H35B	108.9
N2—C18—C20	115.89 (12)	H35A—C35—H35B	107.7
C17—C18—C20	121.45 (12)	H1W—O1W—H2W	107.2 (18)
C16—C19—H19A	109.5		
			
C6—C1—C2—C3	3.01 (18)	C15—C16—C17—C18	−1.2 (2)
C7—C1—C2—C3	−176.05 (11)	C19—C16—C17—C18	177.68 (14)
C6—C1—C2—C13	−169.44 (11)	C14—N2—C18—C17	2.26 (18)
C7—C1—C2—C13	11.50 (17)	C14—N2—C18—C20	−177.50 (11)
C1—C2—C3—C4	1.77 (18)	C16—C17—C18—N2	−0.6 (2)
C13—C2—C3—C4	174.02 (11)	C16—C17—C18—C20	179.14 (13)
C1—C2—C3—C9	−176.97 (11)	C22—N3—C21—C4	−139.10 (13)
C13—C2—C3—C9	−4.72 (18)	C5—C4—C21—N3	−84.62 (14)
C2—C3—C4—C5	−5.90 (18)	C3—C4—C21—N3	90.57 (14)
C9—C3—C4—C5	172.84 (11)	C26—N4—C22—N3	−178.80 (11)
C2—C3—C4—C21	178.94 (11)	C26—N4—C22—C23	−0.68 (18)
C9—C3—C4—C21	−2.32 (18)	C21—N3—C22—N4	−0.42 (19)
C3—C4—C5—C6	5.22 (18)	C21—N3—C22—C23	−178.64 (12)
C21—C4—C5—C6	−179.67 (11)	N4—C22—C23—C24	−0.8 (2)
C3—C4—C5—C11	−173.26 (11)	N3—C22—C23—C24	177.36 (12)
C21—C4—C5—C11	1.85 (18)	C22—C23—C24—C25	1.3 (2)
C2—C1—C6—C5	−3.71 (18)	C22—C23—C24—C27	−178.42 (14)
C7—C1—C6—C5	175.34 (11)	C23—C24—C25—C26	−0.4 (2)
C2—C1—C6—C29	171.98 (11)	C27—C24—C25—C26	179.31 (14)
C7—C1—C6—C29	−8.97 (17)	C22—N4—C26—C25	1.63 (19)
C4—C5—C6—C1	−0.36 (18)	C22—N4—C26—C28	−178.68 (12)
C11—C5—C6—C1	178.12 (11)	C24—C25—C26—N4	−1.1 (2)
C4—C5—C6—C29	−175.88 (11)	C24—C25—C26—C28	179.23 (13)
C11—C5—C6—C29	2.60 (18)	C30—N5—C29—C6	168.54 (10)
C6—C1—C7—C8	−93.07 (14)	C1—C6—C29—N5	−74.81 (14)
C2—C1—C7—C8	85.97 (14)	C5—C6—C29—N5	100.80 (13)
C2—C3—C9—C10	−88.30 (15)	C29—N5—C30—C35	−170.13 (10)
C4—C3—C9—C10	92.98 (15)	C29—N5—C30—C31	71.21 (14)
C4—C5—C11—C12	−94.72 (14)	C29—N5—C30—C34	−48.19 (15)
C6—C5—C11—C12	86.81 (15)	N5—C30—C31—C32	−122.98 (12)
C14—N1—C13—C2	−148.51 (12)	C35—C30—C31—C32	120.53 (13)
C3—C2—C13—N1	−95.00 (13)	C34—C30—C31—C32	3.17 (15)
C1—C2—C13—N1	77.45 (14)	C30—C31—C32—C33	−27.13 (16)
C18—N2—C14—N1	177.18 (11)	C31—C32—C33—C34	40.92 (15)
C18—N2—C14—C15	−2.14 (18)	C32—C33—C34—C30	−38.95 (15)
C13—N1—C14—N2	154.81 (12)	N5—C30—C34—C33	144.32 (12)
C13—N1—C14—C15	−25.87 (18)	C35—C30—C34—C33	−95.53 (13)
N2—C14—C15—C16	0.36 (19)	C31—C30—C34—C33	21.93 (14)
N1—C14—C15—C16	−178.91 (12)	N5—C30—C35—O1	67.44 (13)
C14—C15—C16—C17	1.31 (19)	C31—C30—C35—O1	−173.32 (11)
C14—C15—C16—C19	−177.57 (14)	C34—C30—C35—O1	−58.86 (14)

### {1-[(3,5-Bis{[(4,6-dimethylpyridin-2-yl)amino]methyl}-2,4,6-triethylbenzyl)amino]cyclopentyl}methanol monohydrate (1a) Hydrogen-bond geometry (Å, º)

*Cg*1 and *Cg*2 are the centroids of the C1–C6 and C14–C18/N2 rings, respectively.

**Table:**

*D*—H···*A*	*D*—H	H···*A*	*D*···*A*	*D*—H···*A*
O1*W*—H2*W*···N5	0.87 (1)	1.93 (1)	2.8018 (14)	175 (2)
O1*W*—H1*W*···N2^i^	0.86 (1)	2.03 (1)	2.8780 (15)	166 (2)
O1—H1*A*···O1*W*^i^	0.87 (1)	1.83 (1)	2.6993 (14)	176 (2)
N5—H5···O1*W*^i^	0.90 (1)	2.52 (1)	3.3302 (14)	151 (1)
N3—H3···O1^i^	0.89 (1)	2.05 (1)	2.9115 (15)	162 (2)
N1—H1···O1*W*^i^	0.89 (1)	2.50 (1)	3.2618 (16)	145 (1)
C32—H32*B*···*Cg*1^ii^	0.99	2.69	3.666 (2)	169
C25—H25···*Cg*2^iii^	0.95	2.84	3.728 (2)	156

Symmetry codes: (i) −*x*, −*y*+1, −*z*; (ii) −*x*, −*y*, −*z*; (iii) *x*+1, *y*, *z*.

### {1-[(3,5-Bis{[(4,6-dimethylpyridin-2-yl)amino]methyl}-2,4,6-triethylbenzyl)amino]cyclopentyl}methanol methanol monosolvate (1b) Crystal data

**Table:**

C_35_H_51_N_5_O·CH_4_O	*Z* = 2
*M**_r_* = 589.85	*F*(000) = 644
Triclinic, *P*1	*D*_x_ = 1.046 Mg m^−^^3^
*a* = 12.1169 (4) Å	Mo *K*α radiation, λ = 0.71073 Å
*b* = 13.2380 (5) Å	Cell parameters from 9915 reflections
*c* = 13.6258 (5) Å	θ = 2.8–30.5°
α = 68.373 (2)°	µ = 0.07 mm^−^^1^
β = 79.379 (2)°	*T* = 100 K
γ = 67.392 (2)°	Irregular, colourless
*V* = 1873.32 (12) Å^3^	0.56 × 0.34 × 0.32 mm

### {1-[(3,5-Bis{[(4,6-dimethylpyridin-2-yl)amino]methyl}-2,4,6-triethylbenzyl)amino]cyclopentyl}methanol methanol monosolvate (1b) Data collection

**Table:**

Bruker X8 APEXII CCD diffractometer	*R*_int_ = 0.022
phi and ω scans	θ_max_ = 26.4°, θ_min_ = 1.8°
32191 measured reflections	*h* = −14→15
7654 independent reflections	*k* = −15→16
5160 reflections with *I* > 2σ(*I*)	*l* = 0→17

### {1-[(3,5-Bis{[(4,6-dimethylpyridin-2-yl)amino]methyl}-2,4,6-triethylbenzyl)amino]cyclopentyl}methanol methanol monosolvate (1b) Refinement

**Table:**

Refinement on *F*^2^	0 restraints
Least-squares matrix: full	Hydrogen site location: mixed
*R*[*F*^2^ > 2σ(*F*^2^)] = 0.065	H atoms treated by a mixture of independent and constrained refinement
*wR*(*F*^2^) = 0.184	*w* = 1/[σ^2^(*F*_o_^2^) + (0.026*P*)^2^ + 3.8856*P*] where *P* = (*F*_o_^2^ + 2*F*_c_^2^)/3
*S* = 1.21	(Δ/σ)_max_ < 0.001
7654 reflections	Δρ_max_ = 0.64 e Å^−^^3^
410 parameters	Δρ_min_ = −0.39 e Å^−^^3^

### {1-[(3,5-Bis{[(4,6-dimethylpyridin-2-yl)amino]methyl}-2,4,6-triethylbenzyl)amino]cyclopentyl}methanol methanol monosolvate (1b) Special details

**Table:**

Geometry. All esds (except the esd in the dihedral angle between two l.s. planes) are estimated using the full covariance matrix. The cell esds are taken into account individually in the estimation of esds in distances, angles and torsion angles; correlations between esds in cell parameters are only used when they are defined by crystal symmetry. An approximate (isotropic) treatment of cell esds is used for estimating esds involving l.s. planes.

### {1-[(3,5-Bis{[(4,6-dimethylpyridin-2-yl)amino]methyl}-2,4,6-triethylbenzyl)amino]cyclopentyl}methanol methanol monosolvate (1b) Fractional atomic coordinates and isotropic or equivalent isotropic displacement parameters (Å^2^)

**Table:**

	*x*	*y*	*z*	*U*_iso_*/*U*_eq_	
O1	1.27014 (18)	0.57360 (19)	0.78975 (18)	0.0337 (5)	
H1A	1.3308	0.5159	0.7868	0.051*	
N1	0.8131 (3)	0.6398 (2)	0.68505 (19)	0.0298 (6)	
H1	0.848 (3)	0.588 (3)	0.750 (3)	0.030 (8)*	
N2	0.7667 (3)	0.6725 (3)	0.5157 (2)	0.0382 (7)	
N3	0.6133 (2)	0.73074 (19)	1.09127 (18)	0.0208 (5)	
H3	0.656 (3)	0.667 (3)	1.081 (2)	0.023 (8)*	
N4	0.53696 (19)	0.61277 (18)	1.23326 (17)	0.0182 (4)	
N5	1.12190 (19)	0.67304 (18)	0.94010 (18)	0.0190 (4)	
H5	1.110 (3)	0.628 (3)	0.910 (3)	0.027 (8)*	
C1	0.9042 (2)	0.7808 (2)	0.78008 (19)	0.0159 (5)	
C2	0.7918 (2)	0.7816 (2)	0.76743 (19)	0.0179 (5)	
C3	0.6984 (2)	0.7982 (2)	0.8449 (2)	0.0186 (5)	
C4	0.7184 (2)	0.8192 (2)	0.93271 (19)	0.0173 (5)	
C5	0.8303 (2)	0.8189 (2)	0.94606 (19)	0.0164 (5)	
C6	0.9235 (2)	0.7990 (2)	0.86997 (19)	0.0160 (5)	
C7	1.0021 (2)	0.7657 (2)	0.6940 (2)	0.0207 (5)	
H7A	0.9917	0.7167	0.6589	0.025*	
H7B	1.0809	0.7252	0.7264	0.025*	
C8	1.0008 (3)	0.8814 (2)	0.6114 (2)	0.0287 (6)	
H8A	0.9235	0.9212	0.5781	0.043*	
H8B	1.0652	0.8677	0.5575	0.043*	
H8C	1.0127	0.9296	0.6456	0.043*	
C9	0.5782 (2)	0.7944 (2)	0.8331 (2)	0.0249 (6)	
H9A	0.5417	0.7656	0.9043	0.030*	
H9B	0.5909	0.7389	0.7960	0.030*	
C10	0.4912 (3)	0.9125 (3)	0.7722 (3)	0.0349 (7)	
H10A	0.4798	0.9686	0.8075	0.052*	
H10B	0.4141	0.9056	0.7701	0.052*	
H10C	0.5241	0.9391	0.6999	0.052*	
C11	0.8503 (2)	0.8411 (2)	1.0417 (2)	0.0195 (5)	
H11A	0.9340	0.7959	1.0622	0.023*	
H11B	0.7971	0.8138	1.1016	0.023*	
C12	0.8259 (3)	0.9694 (2)	1.0213 (2)	0.0283 (6)	
H12A	0.8792	0.9967	0.9628	0.042*	
H12B	0.8406	0.9791	1.0851	0.042*	
H12C	0.7425	1.0144	1.0030	0.042*	
C13	0.7709 (3)	0.7627 (2)	0.6698 (2)	0.0240 (6)	
H13A	0.6844	0.7971	0.6565	0.029*	
H13B	0.8140	0.8016	0.6074	0.029*	
C14	0.8157 (3)	0.5977 (3)	0.6068 (2)	0.0301 (7)	
C15	0.8695 (3)	0.4779 (3)	0.6252 (3)	0.0351 (7)	
H15	0.9032	0.4270	0.6910	0.042*	
C16	0.8722 (3)	0.4360 (4)	0.5462 (3)	0.0480 (10)	
C17	0.8216 (4)	0.5129 (4)	0.4515 (3)	0.0548 (12)	
H17	0.8222	0.4854	0.3960	0.066*	
C18	0.7710 (3)	0.6284 (4)	0.4385 (3)	0.0516 (11)	
C19	0.9332 (4)	0.3088 (4)	0.5622 (4)	0.0662 (14)	
H19A	0.8931	0.2853	0.5225	0.099*	
H19B	0.9289	0.2646	0.6375	0.099*	
H19C	1.0172	0.2936	0.5368	0.099*	
C20	0.7149 (5)	0.7156 (5)	0.3377 (3)	0.0755 (16)	
H20A	0.6293	0.7531	0.3520	0.113*	
H20B	0.7252	0.6763	0.2863	0.113*	
H20C	0.7535	0.7741	0.3089	0.113*	
C21	0.6162 (2)	0.8401 (2)	1.0146 (2)	0.0200 (5)	
H21A	0.6267	0.8879	1.0510	0.024*	
H21B	0.5393	0.8828	0.9791	0.024*	
C22	0.5286 (2)	0.7214 (2)	1.17214 (19)	0.0168 (5)	
C23	0.4398 (2)	0.8186 (2)	1.1916 (2)	0.0183 (5)	
H23	0.4344	0.8945	1.1460	0.022*	
C24	0.3604 (2)	0.8028 (2)	1.2778 (2)	0.0206 (5)	
C25	0.3712 (2)	0.6901 (2)	1.3418 (2)	0.0226 (5)	
H25	0.3185	0.6767	1.4022	0.027*	
C26	0.4592 (2)	0.5973 (2)	1.3170 (2)	0.0211 (5)	
C27	0.2644 (3)	0.9050 (2)	1.3014 (2)	0.0293 (6)	
H27A	0.2900	0.9726	1.2716	0.044*	
H27B	0.2505	0.8873	1.3781	0.044*	
H27C	0.1902	0.9218	1.2698	0.044*	
C28	0.4714 (3)	0.4746 (2)	1.3806 (2)	0.0282 (6)	
H28A	0.4429	0.4419	1.3406	0.042*	
H28B	0.4236	0.4724	1.4472	0.042*	
H28C	0.5556	0.4291	1.3956	0.042*	
C29	1.0471 (2)	0.7940 (2)	0.8843 (2)	0.0191 (5)	
H29A	1.0858	0.8231	0.8144	0.023*	
H29B	1.0398	0.8442	0.9257	0.023*	
C30	1.2540 (2)	0.6488 (2)	0.9314 (2)	0.0212 (5)	
C31	1.2823 (2)	0.7329 (2)	0.9663 (2)	0.0277 (6)	
H31A	1.3654	0.7311	0.9441	0.033*	
H31B	1.2264	0.8130	0.9367	0.033*	
C32	1.2660 (3)	0.6884 (3)	1.0860 (3)	0.0313 (7)	
H32A	1.1801	0.7099	1.1090	0.038*	
H32B	1.3061	0.7192	1.1194	0.038*	
C33	1.3256 (3)	0.5566 (3)	1.1131 (2)	0.0308 (6)	
H33A	1.2857	0.5159	1.1770	0.037*	
H33B	1.4113	0.5318	1.1261	0.037*	
C34	1.3113 (2)	0.5300 (2)	1.0158 (2)	0.0265 (6)	
H34A	1.2590	0.4830	1.0346	0.032*	
H34B	1.3901	0.4868	0.9889	0.032*	
C35	1.3038 (2)	0.6496 (2)	0.8197 (2)	0.0270 (6)	
H35A	1.2728	0.7292	0.7694	0.032*	
H35B	1.3922	0.6249	0.8170	0.032*	
O1A	0.95054 (19)	0.4462 (2)	0.85754 (17)	0.0358 (5)	
H1AA	0.9330	0.4031	0.9162	0.054*	
C1A	1.0583 (3)	0.4590 (3)	0.8631 (2)	0.0296 (6)	
H1A1	1.0627	0.5308	0.8087	0.044*	
H1A2	1.1256	0.3929	0.8515	0.044*	
H1A3	1.0623	0.4622	0.9331	0.044*	

### {1-[(3,5-Bis{[(4,6-dimethylpyridin-2-yl)amino]methyl}-2,4,6-triethylbenzyl)amino]cyclopentyl}methanol methanol monosolvate (1b) Atomic displacement parameters (Å^2^)

**Table:**

	*U*^11^	*U*^22^	*U*^33^	*U*^12^	*U*^13^	*U*^23^
O1	0.0203 (10)	0.0337 (12)	0.0483 (13)	0.0011 (9)	0.0023 (9)	−0.0275 (11)
N1	0.0507 (16)	0.0266 (13)	0.0192 (12)	−0.0167 (12)	−0.0054 (11)	−0.0102 (10)
N2	0.0466 (16)	0.0630 (19)	0.0195 (12)	−0.0329 (15)	−0.0007 (11)	−0.0154 (12)
N3	0.0201 (11)	0.0157 (11)	0.0223 (11)	−0.0041 (9)	0.0068 (9)	−0.0074 (9)
N4	0.0167 (10)	0.0180 (10)	0.0200 (11)	−0.0060 (8)	0.0019 (8)	−0.0075 (9)
N5	0.0153 (10)	0.0168 (10)	0.0252 (11)	−0.0034 (8)	−0.0012 (8)	−0.0094 (9)
C1	0.0184 (12)	0.0112 (11)	0.0143 (11)	−0.0024 (9)	0.0019 (9)	−0.0041 (9)
C2	0.0222 (13)	0.0144 (11)	0.0149 (12)	−0.0045 (10)	−0.0027 (10)	−0.0035 (9)
C3	0.0196 (12)	0.0133 (11)	0.0198 (12)	−0.0042 (9)	−0.0008 (10)	−0.0036 (10)
C4	0.0175 (12)	0.0149 (11)	0.0170 (12)	−0.0053 (9)	0.0017 (9)	−0.0039 (9)
C5	0.0186 (12)	0.0140 (11)	0.0151 (11)	−0.0034 (9)	0.0004 (9)	−0.0061 (9)
C6	0.0150 (12)	0.0119 (11)	0.0194 (12)	−0.0028 (9)	−0.0002 (9)	−0.0058 (9)
C7	0.0214 (13)	0.0223 (13)	0.0177 (12)	−0.0066 (10)	0.0038 (10)	−0.0090 (10)
C8	0.0338 (16)	0.0219 (14)	0.0242 (14)	−0.0081 (12)	0.0097 (12)	−0.0076 (11)
C9	0.0213 (13)	0.0315 (15)	0.0254 (14)	−0.0139 (11)	−0.0010 (11)	−0.0082 (12)
C10	0.0247 (15)	0.0402 (18)	0.0346 (17)	−0.0095 (13)	−0.0074 (12)	−0.0057 (14)
C11	0.0220 (13)	0.0199 (13)	0.0175 (12)	−0.0054 (10)	0.0007 (10)	−0.0097 (10)
C12	0.0347 (16)	0.0196 (13)	0.0317 (15)	−0.0048 (12)	−0.0030 (12)	−0.0140 (12)
C13	0.0304 (15)	0.0230 (13)	0.0194 (13)	−0.0114 (11)	−0.0015 (11)	−0.0056 (11)
C14	0.0360 (16)	0.0473 (18)	0.0230 (14)	−0.0286 (14)	0.0079 (12)	−0.0184 (13)
C15	0.0389 (17)	0.0447 (18)	0.0386 (17)	−0.0292 (15)	0.0183 (14)	−0.0265 (15)
C16	0.046 (2)	0.077 (3)	0.056 (2)	−0.045 (2)	0.0341 (18)	−0.053 (2)
C17	0.060 (2)	0.110 (4)	0.044 (2)	−0.065 (3)	0.0349 (18)	−0.060 (2)
C18	0.051 (2)	0.110 (4)	0.0274 (17)	−0.058 (2)	0.0143 (15)	−0.037 (2)
C19	0.066 (3)	0.088 (3)	0.089 (3)	−0.052 (3)	0.052 (2)	−0.078 (3)
C20	0.083 (3)	0.142 (5)	0.030 (2)	−0.066 (3)	0.001 (2)	−0.033 (3)
C21	0.0188 (12)	0.0170 (12)	0.0203 (13)	−0.0051 (10)	0.0046 (10)	−0.0059 (10)
C22	0.0153 (12)	0.0199 (12)	0.0182 (12)	−0.0060 (10)	−0.0002 (9)	−0.0098 (10)
C23	0.0166 (12)	0.0162 (12)	0.0192 (12)	−0.0034 (9)	0.0013 (9)	−0.0061 (10)
C24	0.0185 (12)	0.0226 (13)	0.0210 (13)	−0.0063 (10)	0.0041 (10)	−0.0110 (11)
C25	0.0180 (12)	0.0250 (14)	0.0232 (13)	−0.0073 (11)	0.0057 (10)	−0.0092 (11)
C26	0.0188 (13)	0.0207 (13)	0.0228 (13)	−0.0074 (10)	0.0020 (10)	−0.0069 (11)
C27	0.0268 (14)	0.0202 (13)	0.0314 (15)	−0.0023 (11)	0.0117 (12)	−0.0106 (12)
C28	0.0314 (15)	0.0211 (14)	0.0270 (14)	−0.0098 (12)	0.0087 (12)	−0.0064 (11)
C29	0.0200 (12)	0.0168 (12)	0.0214 (13)	−0.0070 (10)	0.0031 (10)	−0.0085 (10)
C30	0.0147 (12)	0.0198 (13)	0.0309 (14)	−0.0070 (10)	0.0028 (10)	−0.0111 (11)
C31	0.0189 (13)	0.0237 (14)	0.0447 (17)	−0.0077 (11)	−0.0020 (12)	−0.0152 (13)
C32	0.0240 (14)	0.0360 (16)	0.0426 (18)	−0.0095 (12)	−0.0047 (13)	−0.0219 (14)
C33	0.0241 (14)	0.0330 (16)	0.0354 (16)	−0.0110 (12)	−0.0023 (12)	−0.0097 (13)
C34	0.0197 (13)	0.0242 (14)	0.0348 (16)	−0.0058 (11)	0.0008 (11)	−0.0116 (12)
C35	0.0168 (13)	0.0254 (14)	0.0356 (16)	−0.0063 (11)	0.0066 (11)	−0.0114 (12)
O1A	0.0292 (11)	0.0375 (12)	0.0312 (11)	−0.0152 (9)	−0.0019 (9)	0.0033 (9)
C1A	0.0297 (15)	0.0257 (14)	0.0330 (16)	−0.0124 (12)	0.0041 (12)	−0.0089 (12)

### {1-[(3,5-Bis{[(4,6-dimethylpyridin-2-yl)amino]methyl}-2,4,6-triethylbenzyl)amino]cyclopentyl}methanol methanol monosolvate (1b) Geometric parameters (Å, º)

**Table:**

O1—C35	1.423 (4)	C16—C17	1.386 (6)
O1—H1A	0.8400	C16—C19	1.503 (6)
N1—C14	1.364 (4)	C17—C18	1.366 (6)
N1—C13	1.451 (4)	C17—H17	0.9500
N1—H1	0.94 (3)	C18—C20	1.501 (6)
N2—C14	1.337 (4)	C19—H19A	0.9800
N2—C18	1.363 (4)	C19—H19B	0.9800
N3—C22	1.365 (3)	C19—H19C	0.9800
N3—C21	1.448 (3)	C20—H20A	0.9800
N3—H3	0.85 (3)	C20—H20B	0.9800
N4—C22	1.345 (3)	C20—H20C	0.9800
N4—C26	1.346 (3)	C21—H21A	0.9900
N5—C29	1.485 (3)	C21—H21B	0.9900
N5—C30	1.497 (3)	C22—C23	1.402 (3)
N5—H5	0.90 (3)	C23—C24	1.381 (3)
C1—C2	1.400 (4)	C23—H23	0.9500
C1—C6	1.407 (3)	C24—C25	1.391 (4)
C1—C7	1.517 (3)	C24—C27	1.503 (3)
C2—C3	1.408 (3)	C25—C26	1.388 (4)
C2—C13	1.522 (3)	C25—H25	0.9500
C3—C4	1.404 (4)	C26—C28	1.495 (4)
C3—C9	1.517 (4)	C27—H27A	0.9800
C4—C5	1.398 (3)	C27—H27B	0.9800
C4—C21	1.520 (3)	C27—H27C	0.9800
C5—C6	1.401 (3)	C28—H28A	0.9800
C5—C11	1.515 (3)	C28—H28B	0.9800
C6—C29	1.518 (3)	C28—H28C	0.9800
C7—C8	1.526 (4)	C29—H29A	0.9900
C7—H7A	0.9900	C29—H29B	0.9900
C7—H7B	0.9900	C30—C31	1.525 (4)
C8—H8A	0.9800	C30—C35	1.527 (4)
C8—H8B	0.9800	C30—C34	1.549 (4)
C8—H8C	0.9800	C31—C32	1.519 (4)
C9—C10	1.531 (4)	C31—H31A	0.9900
C9—H9A	0.9900	C31—H31B	0.9900
C9—H9B	0.9900	C32—C33	1.536 (4)
C10—H10A	0.9800	C32—H32A	0.9900
C10—H10B	0.9800	C32—H32B	0.9900
C10—H10C	0.9800	C33—C34	1.545 (4)
C11—C12	1.531 (4)	C33—H33A	0.9900
C11—H11A	0.9900	C33—H33B	0.9900
C11—H11B	0.9900	C34—H34A	0.9900
C12—H12A	0.9800	C34—H34B	0.9900
C12—H12B	0.9800	C35—H35A	0.9900
C12—H12C	0.9800	C35—H35B	0.9900
C13—H13A	0.9900	O1A—C1A	1.399 (4)
C13—H13B	0.9900	O1A—H1AA	0.8400
C14—C15	1.406 (5)	C1A—H1A1	0.9800
C15—C16	1.371 (4)	C1A—H1A2	0.9800
C15—H15	0.9500	C1A—H1A3	0.9800
			
C35—O1—H1A	109.5	H19A—C19—H19C	109.5
C14—N1—C13	122.7 (3)	H19B—C19—H19C	109.5
C14—N1—H1	119 (2)	C18—C20—H20A	109.5
C13—N1—H1	118 (2)	C18—C20—H20B	109.5
C14—N2—C18	117.3 (3)	H20A—C20—H20B	109.5
C22—N3—C21	123.8 (2)	C18—C20—H20C	109.5
C22—N3—H3	114 (2)	H20A—C20—H20C	109.5
C21—N3—H3	121 (2)	H20B—C20—H20C	109.5
C22—N4—C26	118.8 (2)	N3—C21—C4	110.2 (2)
C29—N5—C30	115.6 (2)	N3—C21—H21A	109.6
C29—N5—H5	106 (2)	C4—C21—H21A	109.6
C30—N5—H5	106 (2)	N3—C21—H21B	109.6
C2—C1—C6	119.8 (2)	C4—C21—H21B	109.6
C2—C1—C7	119.4 (2)	H21A—C21—H21B	108.1
C6—C1—C7	120.7 (2)	N4—C22—N3	115.6 (2)
C1—C2—C3	120.5 (2)	N4—C22—C23	122.0 (2)
C1—C2—C13	119.7 (2)	N3—C22—C23	122.3 (2)
C3—C2—C13	119.8 (2)	C24—C23—C22	119.2 (2)
C4—C3—C2	118.8 (2)	C24—C23—H23	120.4
C4—C3—C9	120.7 (2)	C22—C23—H23	120.4
C2—C3—C9	120.5 (2)	C23—C24—C25	118.4 (2)
C5—C4—C3	121.1 (2)	C23—C24—C27	120.6 (2)
C5—C4—C21	120.5 (2)	C25—C24—C27	121.0 (2)
C3—C4—C21	118.4 (2)	C26—C25—C24	119.8 (2)
C4—C5—C6	119.6 (2)	C26—C25—H25	120.1
C4—C5—C11	120.3 (2)	C24—C25—H25	120.1
C6—C5—C11	120.1 (2)	N4—C26—C25	121.9 (2)
C5—C6—C1	120.1 (2)	N4—C26—C28	116.3 (2)
C5—C6—C29	120.7 (2)	C25—C26—C28	121.8 (2)
C1—C6—C29	119.2 (2)	C24—C27—H27A	109.5
C1—C7—C8	112.1 (2)	C24—C27—H27B	109.5
C1—C7—H7A	109.2	H27A—C27—H27B	109.5
C8—C7—H7A	109.2	C24—C27—H27C	109.5
C1—C7—H7B	109.2	H27A—C27—H27C	109.5
C8—C7—H7B	109.2	H27B—C27—H27C	109.5
H7A—C7—H7B	107.9	C26—C28—H28A	109.5
C7—C8—H8A	109.5	C26—C28—H28B	109.5
C7—C8—H8B	109.5	H28A—C28—H28B	109.5
H8A—C8—H8B	109.5	C26—C28—H28C	109.5
C7—C8—H8C	109.5	H28A—C28—H28C	109.5
H8A—C8—H8C	109.5	H28B—C28—H28C	109.5
H8B—C8—H8C	109.5	N5—C29—C6	110.1 (2)
C3—C9—C10	113.1 (2)	N5—C29—H29A	109.6
C3—C9—H9A	109.0	C6—C29—H29A	109.6
C10—C9—H9A	109.0	N5—C29—H29B	109.6
C3—C9—H9B	109.0	C6—C29—H29B	109.6
C10—C9—H9B	109.0	H29A—C29—H29B	108.1
H9A—C9—H9B	107.8	N5—C30—C31	110.6 (2)
C9—C10—H10A	109.5	N5—C30—C35	110.3 (2)
C9—C10—H10B	109.5	C31—C30—C35	113.0 (2)
H10A—C10—H10B	109.5	N5—C30—C34	107.1 (2)
C9—C10—H10C	109.5	C31—C30—C34	103.3 (2)
H10A—C10—H10C	109.5	C35—C30—C34	112.1 (2)
H10B—C10—H10C	109.5	C32—C31—C30	103.5 (2)
C5—C11—C12	112.6 (2)	C32—C31—H31A	111.1
C5—C11—H11A	109.1	C30—C31—H31A	111.1
C12—C11—H11A	109.1	C32—C31—H31B	111.1
C5—C11—H11B	109.1	C30—C31—H31B	111.1
C12—C11—H11B	109.1	H31A—C31—H31B	109.0
H11A—C11—H11B	107.8	C31—C32—C33	103.2 (2)
C11—C12—H12A	109.5	C31—C32—H32A	111.1
C11—C12—H12B	109.5	C33—C32—H32A	111.1
H12A—C12—H12B	109.5	C31—C32—H32B	111.1
C11—C12—H12C	109.5	C33—C32—H32B	111.1
H12A—C12—H12C	109.5	H32A—C32—H32B	109.1
H12B—C12—H12C	109.5	C32—C33—C34	105.5 (2)
N1—C13—C2	109.9 (2)	C32—C33—H33A	110.6
N1—C13—H13A	109.7	C34—C33—H33A	110.6
C2—C13—H13A	109.7	C32—C33—H33B	110.6
N1—C13—H13B	109.7	C34—C33—H33B	110.6
C2—C13—H13B	109.7	H33A—C33—H33B	108.8
H13A—C13—H13B	108.2	C33—C34—C30	106.2 (2)
N2—C14—N1	118.4 (3)	C33—C34—H34A	110.5
N2—C14—C15	122.8 (3)	C30—C34—H34A	110.5
N1—C14—C15	118.8 (3)	C33—C34—H34B	110.5
C16—C15—C14	118.6 (4)	C30—C34—H34B	110.5
C16—C15—H15	120.7	H34A—C34—H34B	108.7
C14—C15—H15	120.7	O1—C35—C30	110.5 (2)
C15—C16—C17	119.0 (4)	O1—C35—H35A	109.5
C15—C16—C19	119.8 (4)	C30—C35—H35A	109.5
C17—C16—C19	121.2 (3)	O1—C35—H35B	109.5
C18—C17—C16	119.6 (3)	C30—C35—H35B	109.5
C18—C17—H17	120.2	H35A—C35—H35B	108.1
C16—C17—H17	120.2	C1A—O1A—H1AA	109.5
N2—C18—C17	122.7 (4)	O1A—C1A—H1A1	109.5
N2—C18—C20	115.5 (4)	O1A—C1A—H1A2	109.5
C17—C18—C20	121.8 (4)	H1A1—C1A—H1A2	109.5
C16—C19—H19A	109.5	O1A—C1A—H1A3	109.5
C16—C19—H19B	109.5	H1A1—C1A—H1A3	109.5
H19A—C19—H19B	109.5	H1A2—C1A—H1A3	109.5
C16—C19—H19C	109.5		
			
C6—C1—C2—C3	−1.2 (4)	C15—C16—C17—C18	0.4 (5)
C7—C1—C2—C3	−178.5 (2)	C19—C16—C17—C18	−177.6 (3)
C6—C1—C2—C13	179.5 (2)	C14—N2—C18—C17	0.6 (5)
C7—C1—C2—C13	2.3 (3)	C14—N2—C18—C20	180.0 (3)
C1—C2—C3—C4	2.8 (4)	C16—C17—C18—N2	−0.6 (5)
C13—C2—C3—C4	−177.9 (2)	C16—C17—C18—C20	−180.0 (3)
C1—C2—C3—C9	−177.7 (2)	C22—N3—C21—C4	−177.5 (2)
C13—C2—C3—C9	1.6 (4)	C5—C4—C21—N3	−93.1 (3)
C2—C3—C4—C5	−2.7 (4)	C3—C4—C21—N3	85.7 (3)
C9—C3—C4—C5	177.8 (2)	C26—N4—C22—N3	177.4 (2)
C2—C3—C4—C21	178.5 (2)	C26—N4—C22—C23	−1.5 (4)
C9—C3—C4—C21	−1.0 (3)	C21—N3—C22—N4	177.4 (2)
C3—C4—C5—C6	1.0 (4)	C21—N3—C22—C23	−3.7 (4)
C21—C4—C5—C6	179.7 (2)	N4—C22—C23—C24	1.8 (4)
C3—C4—C5—C11	−179.6 (2)	N3—C22—C23—C24	−177.0 (2)
C21—C4—C5—C11	−0.9 (3)	C22—C23—C24—C25	−0.5 (4)
C4—C5—C6—C1	0.7 (4)	C22—C23—C24—C27	179.7 (3)
C11—C5—C6—C1	−178.7 (2)	C23—C24—C25—C26	−0.9 (4)
C4—C5—C6—C29	−177.8 (2)	C27—C24—C25—C26	178.8 (3)
C11—C5—C6—C29	2.8 (3)	C22—N4—C26—C25	0.0 (4)
C2—C1—C6—C5	−0.6 (3)	C22—N4—C26—C28	178.8 (2)
C7—C1—C6—C5	176.6 (2)	C24—C25—C26—N4	1.3 (4)
C2—C1—C6—C29	178.0 (2)	C24—C25—C26—C28	−177.5 (3)
C7—C1—C6—C29	−4.8 (3)	C30—N5—C29—C6	162.4 (2)
C2—C1—C7—C8	89.8 (3)	C5—C6—C29—N5	90.9 (3)
C6—C1—C7—C8	−87.4 (3)	C1—C6—C29—N5	−87.6 (3)
C4—C3—C9—C10	90.5 (3)	C29—N5—C30—C31	54.1 (3)
C2—C3—C9—C10	−89.0 (3)	C29—N5—C30—C35	−71.7 (3)
C4—C5—C11—C12	−91.6 (3)	C29—N5—C30—C34	166.1 (2)
C6—C5—C11—C12	87.8 (3)	N5—C30—C31—C32	75.2 (3)
C14—N1—C13—C2	−173.9 (3)	C35—C30—C31—C32	−160.5 (2)
C1—C2—C13—N1	83.0 (3)	C34—C30—C31—C32	−39.2 (3)
C3—C2—C13—N1	−96.2 (3)	C30—C31—C32—C33	42.6 (3)
C18—N2—C14—N1	179.7 (3)	C31—C32—C33—C34	−29.1 (3)
C18—N2—C14—C15	−0.4 (4)	C32—C33—C34—C30	5.1 (3)
C13—N1—C14—N2	−6.6 (4)	N5—C30—C34—C33	−96.1 (2)
C13—N1—C14—C15	173.5 (3)	C31—C30—C34—C33	20.7 (3)
N2—C14—C15—C16	0.3 (4)	C35—C30—C34—C33	142.8 (2)
N1—C14—C15—C16	−179.8 (3)	N5—C30—C35—O1	−52.9 (3)
C14—C15—C16—C17	−0.3 (4)	C31—C30—C35—O1	−177.4 (2)
C14—C15—C16—C19	177.8 (3)	C34—C30—C35—O1	66.3 (3)

### {1-[(3,5-Bis{[(4,6-dimethylpyridin-2-yl)amino]methyl}-2,4,6-triethylbenzyl)amino]cyclopentyl}methanol methanol monosolvate (1b) Hydrogen-bond geometry (Å, º)

*Cg*1 and *Cg*3 are the centroids of the C1–C6 and C22–C26/N4 rings, respectively.

**Table:**

*D*—H···*A*	*D*—H	H···*A*	*D*···*A*	*D*—H···*A*
N1—H1···O1*A*	0.94 (3)	2.01 (3)	2.930 (3)	162 (2)
C15—H15···O1*A*	0.95	2.56	3.318 (3)	137
N5—H5···O1	0.90 (3)	2.36 (3)	2.823 (3)	112 (2)
O1—H1*A*···N4^i^	0.84	1.90	2.741 (3)	174
O1*A*—H1*AA*···N5^i^	0.84	1.97	2.798 (3)	170
C27—H27*A*···*Cg*1^ii^	0.98	2.67	3.541 (3)	148
C32—H32*B*···*Cg*3^iii^	0.98	2.69	3.614 (3)	156

Symmetry codes: (i) −*x*+2, −*y*+1, −*z*+2; (ii) −*x*+1, −*y*+2, −*z*+2; (iii) *x*+1, *y*, *z*.
